# A biphasic role of non-canonical Wnt16 signaling during early anterior-posterior patterning and morphogenesis of the sea urchin embryo

**DOI:** 10.1242/dev.168799

**Published:** 2019-12-16

**Authors:** Marina Martínez-Bartolomé, Ryan C. Range

**Affiliations:** Department of Biological Sciences, Auburn University, Auburn, AL 36849, USA

**Keywords:** Wnt signal transduction, Anterior-posterior, Deuterostome evolution, Gene regulatory networks, Wnt16, Frizzled

## Abstract

A Wnt signaling network governs early anterior-posterior (AP) specification and patterning of the deuterostome sea urchin embryo. We have previously shown that non-canonical Fzl1/2/7 signaling antagonizes the progressive posterior-to-anterior downregulation of the anterior neuroectoderm (ANE) gene regulatory network (GRN) by canonical Wnt/β-catenin and non-canonical Wnt1/Wnt8-Fzl5/8-JNK signaling. This study focuses on the non-canonical function of the Wnt16 ligand during early AP specification and patterning. Maternally supplied *wnt16* is expressed ubiquitously during cleavage and zygotic *wnt16* expression is concentrated in the endoderm/mesoderm beginning at mid-blastula stage. Wnt16 antagonizes the ANE restriction mechanism and this activity depends on a functional Fzl1/2/7 receptor. Our results also show that zygotic *wnt16* expression depends on both Fzl5/8 and Wnt/β-catenin signaling. Furthermore, Wnt16 is necessary for the activation and/or maintenance of key regulatory endoderm/mesoderm genes and is essential for gastrulation. Together, our data show that Wnt16 has two functions during early AP specification and patterning: (1) an initial role activating the Fzl1/2/7 pathway that antagonizes the ANE restriction mechanism; and (2) a subsequent function in activating key endoderm GRN factors and the morphogenetic movements of gastrulation.

## Abstract (precis)

**Summary:** Non-canonical Wnt16 signaling is essential for establishing the position of the early germ layer gene regulatory networks along the anterior-posterior axis, and activates molecular mechanisms necessary for gastrulation and mesenchyme morphogenesis.

## INTRODUCTION

In metazoans, anterior-posterior (AP) axis specification and patterning is one of the first fundamental developmental processes that is crucial for establishing the correct adult body plan. Studies have shown a highly conserved role for Wnt signaling in establishing the AP axis in a number of deuterostome species, from sea urchins to mammals (Loh et al., 2016; Niehrs, 2010; Petersen and Reddien, 2009). In these animals, high posterior ‘canonical’ Wnt/β-catenin signaling is necessary to establish the endomesoderm gene regulatory network (GRN) around the posterior pole (Loh et al., 2016; Niehrs, 2010; Petersen and Reddien, 2009). In addition, a mechanism that depends on posterior Wnt/β-catenin is required to restrict the initial broadly expressed anterior neuroectoderm (ANE) GRN to a domain around the anterior pole (Angerer et al., 2011; Gaspard et al., 2008; Kiecker and Niehrs, 2001; Lekven et al., 2001; Nordström et al., 2002; Pani et al., 2012; Yaguchi et al., 2008). Importantly, core members of both the endomesoderm and ANE GRNs have been shown to be conserved in several metazoan taxa (Croce et al., 2006a; Davidson and Erwin, 2006; Hinman and Cheatle Jarvela, 2014; Loh et al., 2016; Wei et al., 2016).

In addition to the Wnt/β-catenin signaling pathway, two conserved ‘alternative/non-canonical’ Wnt signaling pathways, Wnt/JNK and Wnt/Ca^2+^, have been identified in a variety of metazoan embryos. Recent studies have shown that in many cases during development and adult tissue homeostasis, two or more of these three pathways are active in the same cells or territories (Kestler and Kühl, 2008; van Amerongen and Nusse, 2009). These Wnt signaling networks are necessary for various cellular processes, such as cell fate specification, neurogenesis, spindle orientation and maintenance of stem cells (Kestler and Kühl, 2008; van Amerongen and Nusse, 2009). Our lab has discovered that AP specification and patterning in sea urchin embryos depend on integrated cross-regulatory information from all three major Wnt signaling branches (Khadka et al., 2018; Range, 2014, 2018; Range and Wei, 2016; Range et al., 2013), making this one of the few known examples where all three Wnt signaling pathways have been shown to govern a fundamental developmental process. The sea urchin AP axis is initiated when maternally localized Wnt signaling components (e.g. Dishevelled) promote nuclear accumulation of β-catenin around the 16-cell stage, resulting in the activation of the endomesoderm GRN (Davidson et al., 2002; Logan et al., 1999; Weitzel et al., 2004; Wikramanayake et al., 1998; Wikramanayake et al., 2003; Wikramanayake et al., 2004). In addition, nuclear β-catenin is necessary to repress the activity of an unknown, broadly expressed regulatory network that drives ubiquitous activity of the ANE GRN, restricting its expression to the anterior half of the embryo at the 32-cell stage (Wei et al., 2009; Yaguchi et al., 2008). Around the same time, Wnt/β-catenin signaling activates the Wnt1 and Wnt8 ligands in posterior cells, which appear to diffuse anteriorly where they interact with the Wnt receptor Fzl5/8 and activate the intracellular messenger JNK in equatorial ectoderm (an ectoderm territory established between the posterior endomesoderm GRN and the anterior ANE GRN around by mesenchyme blastula stage). The result of Wnt1/Wnt8-Fzl5/8-JNK signaling is the progressive posterior-to-anterior downregulation of ANE GRN activity in ectodermal cells during the late cleavage and blastula stages, ultimately restricting the ANE to a territory around the anterior pole (Range, 2018; Range et al., 2013). During the final phase of this ANE restriction (late blastula/early gastrula stages), the combined activity of the secreted Wnt modulators sFRP-1, sFRP1/5, Dkk3 and Dkk1 around the anterior pole establishes the correctly sized ANE territory (Khadka et al., 2018; Range and Wei, 2016; Range et al., 2013). Importantly, non-canonical Fzl1/2/7 signaling represents a third Wnt signaling branch in this process and is essential during the entire progressive posterior-to-anterior ANE restriction mechanism. This pathway antagonizes the activity of both the Wnt/β-catenin and Fzl5/8-JNK pathways, governing the rate of ANE restriction (Range, 2018; Range et al., 2013) and allows for the establishment of four early regulatory networks along the AP axis by early gastrulation: posterior endoderm and mesoderm GRNs, an equatorial ectoderm GRN and the ANE GRN around the anterior pole (see Fig. 8A for model). Interestingly, expression and/or functional studies in other deuterostome embryos strongly suggest that major aspects of the AP Wnt signaling network identified in sea urchin embryos may be conserved among deuterostome embryos (Range, 2014).

A combination of the activities of secreted Wnt modulators, trans-membrane Frizzled receptors and co-receptors is necessary in order to activate one or more Wnt signaling pathways in a given context. It is arguably the spatiotemporal expression of the Frizzled receptors and the particular Wnt ligand(s) they interact with that is the primary determinant of which Wnt signaling branch will be activated, and at what levels these pathways will be activated, in a given cell or tissue territory. Previous studies in sea urchin embryos have defined the composition and spatiotemporal expression patterns of the Wnt ligands and Frizzled receptors, including a set of four Frizzleds and 11 or 12 Wnts, depending on the species (Croce et al., 2006b; Robert et al., 2014, 2019). Fzl5/8 and Fzl1/2/7 are the only receptors expressed during the early specification and patterning of the AP axis by the Wnt signaling network (0 to 24 hpf) (Croce et al., 2006b; Lhomond et al., 2012; Range, 2018; Range et al., 2013). Both genes are expressed ubiquitously during cleavage and early blastula stages, and then progressively restricted to more anterior cells by late blastula stages (Croce et al., 2006b; Lhomond et al., 2012). In addition, six Wnt ligands are expressed during this process (Wnt1, Wnt5, Wnt6, Wnt7, Wnt8 and Wnt16), whereas the other five or six ligands are expressed at or after late blastula/early gastrula stages, i.e. after the completion of early AP patterning (Croce et al., 2006b; Robert et al., 2014, 2019). As mentioned above, we have previously demonstrated that two of these six ligands, Wnt1 and Wnt8, activate Fzl5/8-JNK signaling (Range et al., 2013); however, the secreted Wnt ligand(s) required for activation of non-canonical Fzl1/2/7 signaling and antagonism of Wnt1/Wnt8-Fzl5/8-JNK signaling have not been identified. Fzl1/2/7 signaling appears to be active as early as the 32-cell stage throughout the embryo, suggesting that the Wnt ligand(s) that activate it should also be broadly expressed at or before the 32-cell stage. Of the remaining four ligands that could potentially activate Fzl1/2/7, *wnt6*, *wnt7* and *wnt16* are maternally supplied (Croce et al., 2011; Robert et al., 2014), and only one of these, *wnt7*, has been shown to be expressed ubiquitously during cleavage and blastula stages. In contrast, *wnt5* expression is activated around the 60- to 120-cell stage when it is expressed in cells at the boundary between the endoderm and ectoderm, suggesting that it does not play a role in the antagonism of Wnt/β-catenin and Fzl5/8 signaling throughout the embryo at the 32-cell stage (Cui et al., 2014; McIntyre et al., 2013; Robert et al., 2014). Consistent with this idea, functional studies indicate that Wnt5 acts as a short-range signal necessary for specification of cells along the border ectoderm, not for broad Fzl1/2/7 signaling (McIntyre et al., 2013). Functional analyses also show that Wnt6 is necessary for the activation and/or maintenance of the endomesoderm GRN mediated by Wnt/β-catenin during blastula stages, and that overexpression of Wnt6 promotes endomesoderm gene expression throughout the embryo (Croce et al., 2011; Lhomond et al., 2012). These results are inconsistent with a role for Wnt6 in the Fzl1/2/7 signaling-mediated antagonism of Wnt/β-catenin and/or Fzl5/8-JNK signaling during early AP patterning.

In this study, we show that activation of the non-canonical Fzl1/2/7 signaling pathway during cleavage and blastula stages requires Wnt16 activity. Knockdown of Wnt16-Fzl1/2/7 activity allows the Wnt1/Wnt8-Fzl5/8-JNK pathway to eliminate the ANE GRN from around the anterior pole from the beginning of ANE restriction mechanism. We further show that two Wnt network signaling pathways, Wnt/β-catenin and Fzl5/8-JNK, control zygotic *wnt16* expression in the endoderm and mesoderm during late blastula and gastrula stages. At this time, our data indicate that Wnt16 signaling has a temporally distinct second role during early embryogenesis in regulating the expression of crucial endoderm and mesoderm GRNs components, and the morphogenetic movements of gastrulation.

## RESULTS

### The spatiotemporal expression of *wnt16* during early AP specification and patterning

**Fig. 1.**
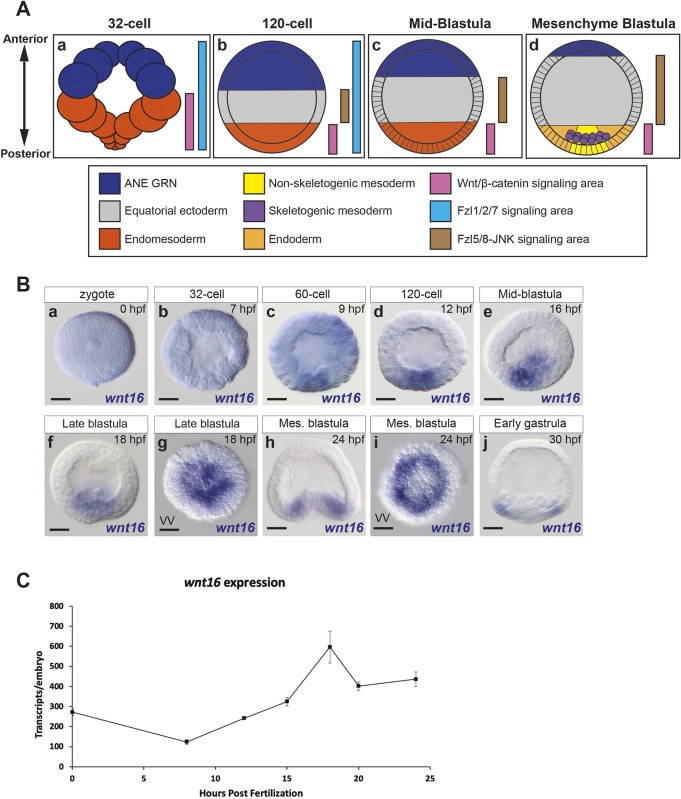
**Spatiotemporal expression of the *wnt16* ligand during early anterior-posterior specification and patterning.** (A) Diagram showing a model for the areas of Wnt/β-catenin, Fzl5/8-JNK and Fzl1/2/7 signaling during ANE early AP patterning, consistent with previous data (Khadka et al., 2018; Range, 2018; Range and Wei, 2017; and Range et al., 2013). (B) Whole-mount *in situ* hybridization analysis of *wnt16* expression during ANE restriction. (Ba,b) Expression of *wnt16* mRNA transcripts was first detected and they were broadly expressed throughout zygotes and 32-cell-stage embryos. (Bd-j) Between the 120-cell stage and late gastrula (30 hpf), *wnt16* expression was progressively downregulated from anterior and equatorial ectoderm cells, resulting in a localized expression in the posterior endoderm and mesoderm region of the embryo. (C) qPCR measurements showing the temporal expression of *wnt16* transcripts from three different batches of embryos from egg to mesenchyme blastula stages (24 hpf). The number of *wnt16* transcripts per embryo (*y* axis) is based on the Ct value of *z12* transcripts. The absolute concentrations of *z12* transcripts are known at each stage (Wang et al., 1995). Scale bars: 20 µm. VV, vegetal view.

Ubiquitously expressed *wnt16* mRNA is the most abundant maternal Wnt mRNA during early cleavage stages in sea urchin embryos (Stamateris et al., 2010). Although the early spatial expression of *wnt16* had previously been examined in the sea urchin embryo (Stamateris et al., 2010), here we present a more detailed *wnt16* expression pattern analysis. We performed whole-mount *in situ* hybridization with *wnt16* antisense probe during the early stages of AP specification and patterning. Transcripts were ubiquitously expressed from the zygote and broad expression was detected until the 32-cell stage (Fig. 1Ba,b). Beginning at the 60-cell stage, an enrichment of *wnt16* transcripts was detected in posterior endomesoderm cells (Fig. 1Bc-e). Around the mid-blastula stage, the expression levels of *wnt16* transcripts decreased in anterior cells and were strongly upregulated in the endoderm and mesodermal cell territories in blastula and gastrula stage embryos [18-30 h post fertilization (hpf) in *Strongylocentrotus purpuratus*] (Fig. 1Bf-j). Vegetal views of late blastula stage embryos showed that *wnt16* was expressed broadly throughout posterior/vegetal cells; by mesenchyme blastula stages, *wnt16* expression was distributed in a concentric ring around the posterior pole (Fig. 1Bg,Bi). Consistent with the whole-mount *in situ* hybridization data, qPCR analysis at the same developmental time points showed low levels of *wnt16* transcripts in the zygote and at the 32-cell stage (Fig. 1C), then the level of transcripts decreased until zygotic mechanisms activated *wnt16* expression during the blastula stages, reaching maximal levels by the mesenchyme blastula/early gastrula stages (Fig. 1C). These data indicate that *wnt16* is expressed in a spatiotemporal pattern, consistent with an early role in the early AP Wnt network, possibly as an activator of the Fzl1/2/7 signaling pathway. Additionally, *wnt16* expression in the posterior endomesoderm cells at mesenchyme blastula stage suggests that this Wnt ligand could also be involved in the specification of the endomesoderm territory and/or the morphogenetic movements of gastrulation.

### Wnt16 is necessary to repress early endomesoderm gene expression and for the specification of the ANE territory

**Fig. 2.**
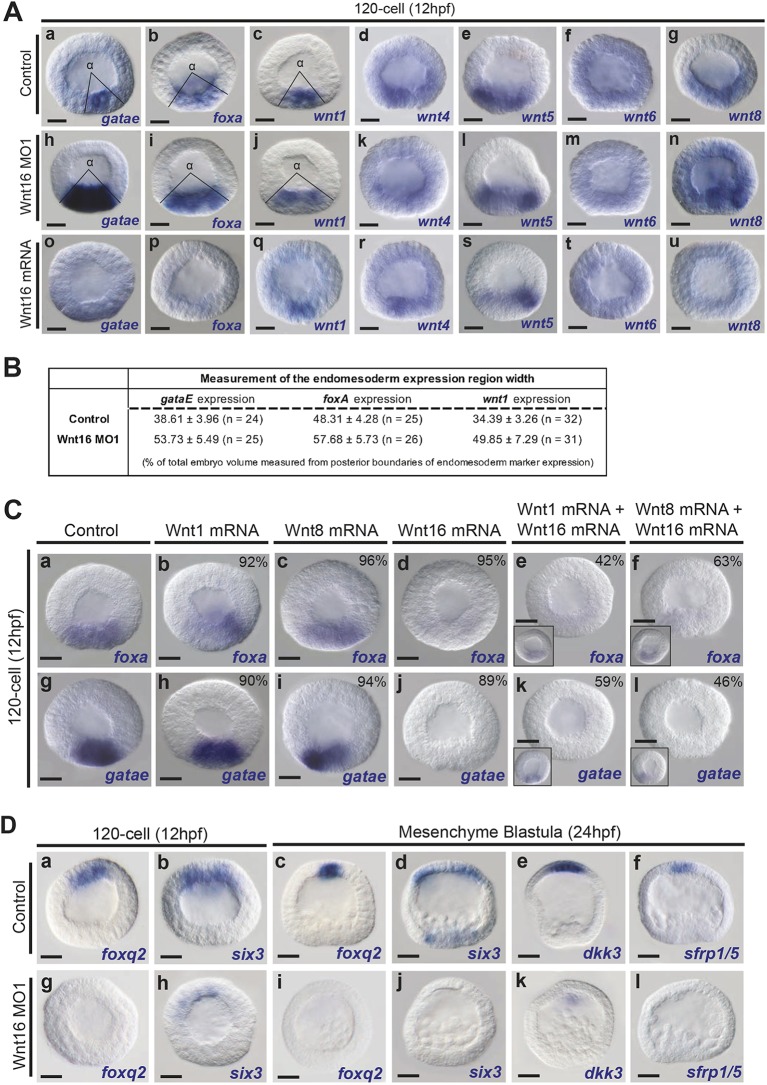
**Wnt16 represses endomesoderm genes at the 120-cell stage and is necessary for ANE GRN expression.** (A) Expression of the endomesoderm markers *gataE*, *foxA*, *wnt1* and *wnt8*, and three Wnt ligands (*wn4*, *wnt5* and *wnt6*) in control, Wnt16 morpholino-injected and *wnt16* mRNA-injected embryos at the 120-cell stage. The expression of the endomesoderm markers *gataE*, *foxA*, *wnt1* and *wnt8* was upregulated in Wnt16 MO1-injected embryos (Ah-j,n compared with Aa-c,g). *wnt16* mRNA overexpression downregulated the expression of the endomesoderm markers *gataE*, *foxA*, *wnt1* and *wnt8* (Ah-j,n compared with Aa-c,g). Solid lines indicate the posterior boundaries of each endomesoderm marker. The expression of *wnt4*, *wnt5* and *wnt6* ligands was not affected in Wnt16 morpholino-injected and *wnt16* mRNA-injected embryos (Ak-m,r-t compared with Ad-f). (B) The angle α shown in A was measured from three different samples of 120-cell-stage embryos using ImageJ. Volume=0.5(1-cos α/2) was used to calculate the percentage of the surface area (±s.e.m.) occupied by the endomesoderm territories in control and Wnt16 knockdowns. (C) Wnt1 and/or Wnt8 might interact with Wnt16 to repress endomesoderm genes at the 120-cell stage. The expression of the endomesoderm markers *foxA* and *gataE* was not affected in *wnt1* or *wnt8* mRNA-injected embryos (Cb,c and Ch,i) compared with control (Ca,g). In embryos overexpressing *wnt16* mRNA, *foxA* and *gataE*, expression was severely downregulated (Cd,j). Embryos co-injected with *wnt16* mRNA and either *wnt1* or *wnt8* mRNA showed either a strong downregulation (main panels) or a significantly reduced expression (insets) of both *foxA* and *gataE* genes (Ce,f and Ck,l). Insets show significantly reduced phenotypes of *foxA* and *gataE* expression in embryos injected with *wnt1* and *wnt16* mRNA (40% and 33%, respectively), and in embryos injected with *wnt8* and *wnt16* mRNA (33% and 43%). The remaining percentages of embryos observed that show the representative phenotypes depicted are indicated in each panel. (D) *foxq2* and *six3* expression at the 120-cell stage in control (Da,b) and Wnt16 morpholino-injected embryos (Dg,h). Expression of ANE makers (*foxq2*, *six3*, *dkk3* and *sfrp1/5*) in control embryos (Dc-f) and in Wnt16 MO1-injected embryos (Di-l) at the mesenchyme blastula stage (24 hpf). MO, morpholino. Scale bars: 20 µm.

In a previous study, we showed that Fzl1/2/7 signaling acts broadly throughout early cleavage/blastula stage embryos as an antagonistic buffer to early posterior Wnt/β-catenin signaling. In the absence of Fzl1/2/7 activity, nuclear β-catenin transcriptional activity was upregulated by ∼2.5 fold and the expression of *wnt8* expanded towards the anterior of the embryo (Range et al., 2013). Based on the broad, early expression pattern of *wnt16*, we hypothesized that Wnt16 might activate Fzl1/2/7 signaling at this time. To test this idea, we used two different morpholino oligonucleotides to perturb Wnt16 function and examined the expression of several core endomesodermal regulatory genes known to be activated by posterior/vegetal Wnt/β-catenin signaling (Emily-Fenouil et al., 1998; Logan et al., 1999; Sethi et al., 2012; Wikramanayake et al., 1998). At the 120-cell stage, the expression of the endomesoderm markers *gataE*, *foxA*, *wnt1* and *wnt8* was upregulated in Wnt16 morphants (Fig. 2Ah-j,n compared with Aa-c,g). Angle measurements of the surface area occupied by gene expression confirmed the upregulation of these endomesoderm genes in Wnt16 morpholino-injected embryos (Fig. 2B). Angle measurements were not performed for *wnt8* expression in Wnt16 knockdowns as it was expressed broadly throughout the embryo (Fig. 2An compared with Ag). Additionally, qPCR analysis confirmed the upregulation of these endomesoderm genes in Wnt16 morphants at 12 hpf (Fig. S2A). In contrast, embryos injected with *wnt16* mRNA showed reduced expression of these endomesoderm genes (Fig. 2Ao-q,u).

*wnt16* expression suggested that it could regulate the expression of the other Wnt ligands expressed during early cleavage stages (*wnt4, wnt5* and *wnt6*). However, manipulating Wnt16 expression did not affect their expression (Fig. 2Ak-m,r-t compared with Ad-f). Together, these data suggest that Wnt16 is necessary to repress posterior endomesoderm GRN activated by Wnt/β-catenin signaling but does not affect the expression of other Wnt ligands during early cleavage stages.

Previous data suggest that the initial nuclear accumulation of β-catenin in posterior blastomeres is ligand independent (Cui et al., 2014; Peng and Wikramanayake, 2013). However, Wnt16 could act in a dominant-negative manner to compete with other Wnt ligands during cleavage stages, preventing Wnt ligand/Fzl receptor interactions that inhibit early Wnt/β-catenin signaling mediated endomesoderm GRN expression. To test this hypothesis, we examined possible interactions among Wnt1, Wnt8 and Wnt16 at the 120-cell stage (Fig. 2C), as Wnt1 and Wnt8 have been implicated in endomesoderm specification during blastula/gastrula stages (Sethi, et al., 2012; Wikramanayake et al., 2004). As expected, overexpression of Wnt1 and Wnt8 did not upregulate the expression of the endomesoderm genes *foxA* and *gataE* at the 120-cell stage (compare Fig. 2Ca,g versus 2Cb,c,h,i), whereas their expression was eliminated in *wnt16* mRNA-injected embryos (*foxA*, 95%; *n*=60/63; Fig. 2Cd) (*gataE*, 89%; *n*=72/81; Fig. 2Cj). In embryos co-injected with *wnt16* mRNA and either *wnt1* or *wnt8* mRNA the expression of these genes was also strongly downregulated in a majority of embryos (Fig. 2Ce,f,k,l). Interestingly, some co-injected embryos showed faint expression of *foxA* and *gataE* compared with embryos injected only with *wnt16* mRNA (Fig. 2Ce,f,k,l, small panels). As previously reported, *foxq2* expression was severely downregulated in embryos injected with *wnt1* mRNA (*n*=60/66) (Fig. S4B) and *wnt8* mRNA-injected embryos (*n*=70/80) (Fig. S4C) at 120-cell stage compared with control embryos (Fig. S4A). In the large majority of embryos, co-injections of *wnt16* mRNA together with *wnt1* mRNA (*n*=80/94) (Fig. S4E) or *wnt8* mRNA (*n*=65/72) (Fig. S4F) did not rescue *foxq2* expression. Taken together, our data indicate that Wnt16 does not interfere with the nuclear accumulation of β-catenin by interfering with Wnt1 or Wnt8, but that high levels of Wnt1 and Wnt8 could slightly interfere with the ability of Wnt16 to activate the Fzl1/2/7 receptor.

Fzl1/2/7 signaling also antagonizes the ANE restriction mechanism mediated by Wnt/β-catenin in posterior cells and Wnt1/Wnt8-Fzl5/8-JNK signaling in anterior ectoderm cells. If Fzl1/2/7 signaling is blocked, these pathways appear to precociously eliminate the ANE GRN as early as the 32- to 60-cell stage (Range et al., 2013). Thus, we examined the expression of the two earliest genes known to be activated in the ANE GRN, *six3* and *foxq2*, in Wnt16 knockdown embryos during early cleavage stages. The expression of both genes was downregulated in 120-cell-stage embryos (Fig. 2Dg-h compared with Da-b), suggesting that Wnt16 is necessary for their early activation. Similarly, the expression of ANE GRN genes around the anterior pole at the end of ANE restriction (mesenchyme blastula stage/24 hpf) depended on Wnt16 function (Fig. 2Dc-f versus Di-l). Consistent with the whole-mount *in situ* hybridization data, qPCR analysis showed that the transcripts levels per embryo for genes in the 24 hpf ANE regulatory network were consistently downregulated in mesenchyme blastula embryos injected with Wnt16 morpholinos (Fig. S1B). In contrast to these results, embryos injected with Wnt7 morpholino expressed *foxq2*, indicating that it is not necessary for ANE specification (Fig. S3C). Together, these data indicate that Wnt16 is necessary from the earliest stages for specification of the ANE territory and for antagonizing the ANE restriction mechanism.

### Wnt16-Fzl1/2/7 signaling is required to antagonize the Wnt1/Wnt8-Fzl5/8-JNK-mediated ANE restriction mechanism

**Fig. 3.**
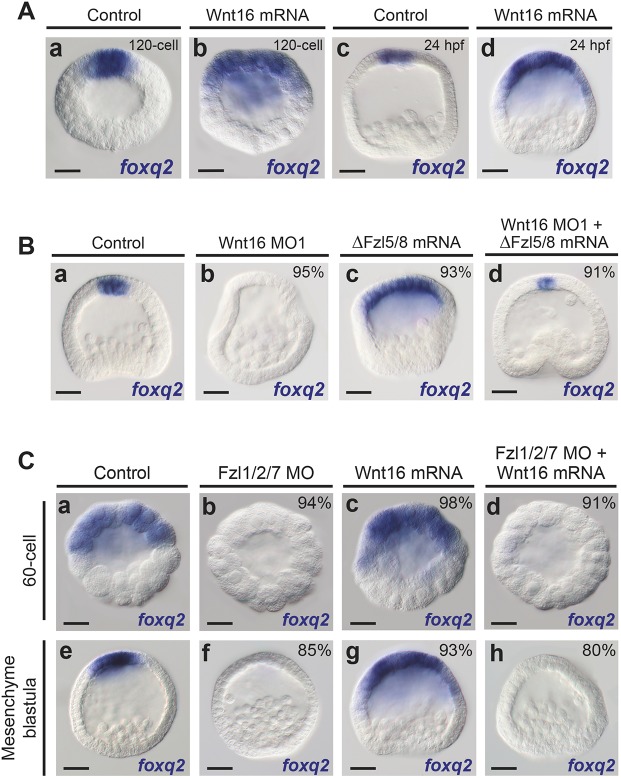
**Wnt16-Fzl1/2/7 signaling antagonizes the Wnt1/Wnt8-Fzl5/8-JNK pathway during the ANE restriction mechanism.** (A) The expression of the ANE marker *foxq2* expanded in embryos overexpressing *wnt16* mRNA at the mesenchyme blastula stage (compare Ab,d with Ab,c). (B) At mesenchyme blastula stage, the expression of the cardinal regulator *foxq2* was expanded in ΔFzl5/8 mRNA-injected embryos (Bc) compared with control embryos (Ba). In the absence of Wnt16, *foxq2* expression was severely downregulated in ANE (Bb), whereas Wnt16 morphants co-injected with ΔFzl5/8 rescued the expression of ANE factors, showing a normal or expanded *foxq2* expression (91%) (Bd). (C) Control embryos showing *foxq2* expression at the 120-cell and mesenchyme blastula stage (24 hpf) (Ca,Ce). ANE expression of *foxq2* was completely eliminated in Fzl1/2/7 morpholino-injected embryos (Cb,Cf). At the 120-cell stage, *foxq2* was expressed in embryos injected with *wnt16* mRNA (Cc). At mesenchyme blastula stage, *foxq2* expression was strongly upregulated and expanded towards the posterior pole in embryos injected with *wnt16* mRNA (Cg). Overexpression of *wnt16* in a Fzl1/2/7 morphant background produced a completely elimination of *foxq2* expression, mimicking the Fzl1/2/7 knockdown phenotype (Cd,Ch). MO, morpholino; ΔFzl5/8, dominant negative Fzl5/8. Scale bars: 20 µm.

We reasoned that overexpression of Wnt16 should antagonize the ANE restriction mediated by Wnt/β-catenin and Wnt1/Wnt8-Fzl5/8-JNK signaling if the Wnt16 ligand is necessary to activate Fzl1/2/7 signaling. Consistent with this idea, expression of the ANE cardinal regulator *foxq2* was expanded throughout the anterior hemisphere in mesenchyme blastula stage embryos injected with *wnt16* mRNA (Fig. 3A). This expansion of ANE factors is remarkably similar to expanded *foxq2* expression observed in embryos lacking functional Fzl5/8-JNK signaling (Khadka et al., 2017; Range et al., 2013; Range and Wei, 2016; Range, 2018). Next, we asked whether blocking Fzl5/8 function could rescue ANE expression in embryos lacking Wnt16. In three different batches of embryos, we injected one set of zygotes with mRNA encoding a dominant-negative form of the Fzl5/8 receptor (ΔFzl5/8) (Croce et al., 2006b; Range et al., 2013) (Fig. 3Bc), a second set with Wnt16 MO1 (Fig. 3Bb), and a third with Wnt16 MO1 and ΔFzl5/8 (Fig. 3Bd). When we blocked the function of Fzl5/8 by injecting ΔFzl5/8, the expression of *foxq2* was expanded in the anterior half of mesenchyme blastula embryos, confirming our previous report (Range et al., 2013) (Fig. 3Bc). As shown above, *foxq2* expression was undetectable in 95% of embryos lacking Wnt16 (*n*=70/74; Fig. 3Bb). In contrast, the large majority of zygotes doubly injected with Wnt16 MO1 and ΔFzl5/8 mRNA showed rescue of *foxq2* expression (91% rescue; *n*=63/69; Fig. 3Bd). These results indicate that Wnt16 activity antagonizes Wnt1/Wnt8-Fzl5/8-JNK signaling during the ANE restriction process, allowing the proper size of the final ANE territory.

In addition, we studied the epistatic relationship between Wnt16 and Fzl1/2/7 receptor to investigate whether the Wnt16 ligand functions to activate the Fzl1/2/7 signaling pathway. Similar to the above experiments, in three different batches of embryos, we injected one set of zygotes with *wnt16* mRNA, another set with Fzl1/2/7 morpholino, and a third set with both *wnt16* mRNA and Fzl1/2/7 morpholino (Fig. 3C). At the 32-cell stage, the ability of overexpressed *wnt16* to inhibit the restriction mechanism cannot be assessed, but *foxq2* was expressed in 98% of these embryos (*n*=64/65; Fig. 3Cc). In contrast, *foxq2* expression was completely eliminated in 94% of Fzl1/2/7 morphants (*n*=58/62; Fig. 3Cb), as well as in 91% of the embryos injected with both Fzl1/2/7 morpholino and *wnt16* mRNA (*n*=64/70; Fig. 3Cd). The expression of *foxq2* was expanded broadly in the ectoderm of mesenchyme blastula stage embryos (24 hpf) injected with mRNA encoding *wnt16* (93%; *n*=71/76; Fig. 3Cg compared with Ce), but its expression was eliminated in Fzl1/2/7 knockdown embryos (85%; *n*=55/65; Fig. 3Cf) and in embryos co-injected with *wnt16* mRNA and Fzl1/2/7 morpholino (80%; *n*=57/71; Fig. 3Ch). These results strongly support the conclusion from Wnt16 loss-of-function analyses that the Fzl1/2/7-dependent antagonism of Fzl5/8-mediated ANE restriction is activated by the Wnt16 ligand.

### Dynamic change in gene expression patterns of *wnt16*, *eve*, *foxA* and *gcm* during early endoderm and mesoderm patterning

**Fig. 4.**
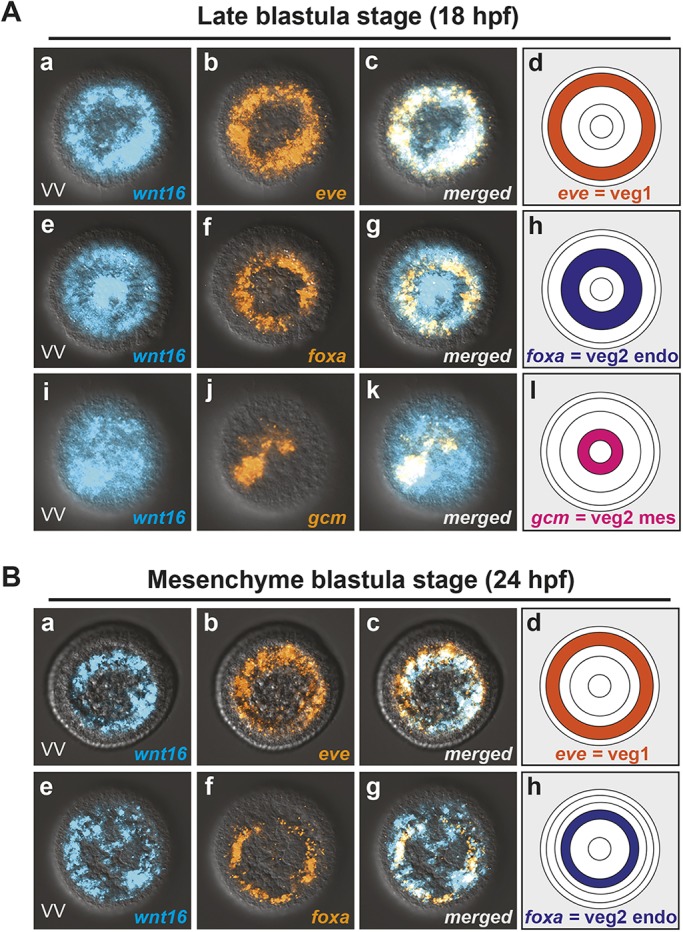
**Gene expression patterns of *wnt16*, eve, *foxA* and *gcm*.** (A) Double *in situ* hybridization showing posterior/vegetal views that combine *wnt16* and either *eve* (Aa-c)*, foxA (*Ae-g) or *gcm* (Ai-k) at late blastula stage (18 hpf). (Ad,h,l) Schematic diagrams showing spatial expression patterns in relation to cell lineage (anterior/veg1 endoderm, posterior/veg2 endoderm and veg2 mesoderm). (B) Posterior/vegetal views that combine *wnt16* and either *eve* (Ba-c) or *foxA* (Ae-g) at mesenchyme blastula stage (24 hpf). (Bd,h) Schematics showing spatial patterns of gene expression in relation to cell linage. endo, endoderm precursors; mes, mesoderm precursors.

While the zygotic spatiotemporal expression pattern of zygotic *wnt16* has been reported elsewhere (Cui et al., 2014; Robert et al., 2014; Stamateris et al., 2010), it is still unclear in which endoderm and mesoderm cells it is expressed leading up to gastrulation. To better understand the molecular signature of Wnt16 during these stages, we analyzed the spatiotemporal expression relationship between *wnt16* and three well characterized genes in the endoderm and mesoderm GRNs (*eve*, *foxA* and *gcm*). As previously reported by Peter and Davidson, 2011, by late blastula (18 hpf) and mesenchyme blastula (24 hpf) stages, the endoderm and mesoderm territories have segregated. At these times, *eve* expression defines the anterior-most Veg1 ring of endoderm cells (Fig. 4Ab,d,Bb,d), *fork-head box A* (*foxA*) defines the more posterior endoderm Veg2 ring of cells (Fig. 4Af,h,Bf,h), and mesoderm regulatory gene *glial cells missing* (*gcm*) expression is detected in the inner Veg2 ring (Fig. 4Aj,l). Here, we show that *wnt16* was co-expressed with *eve*, *foxA* and *gcm* at late blastula stage, indicating that it is expressed throughout the endoderm and mesoderm territories (Fig. 4Ac,g,k). By mesenchyme blastula stage, *wnt16* was downregulated around the most posterior Veg2 mesoderm region but was expressed in a ring of cells overlapping both *eve* and *foxA* expression, indicating that it was transcribed in both the Veg1 and Veg2 endoderm cells at this time (Fig. 4Bc,g). These results suggest that Wnt16 may have a role in inducing and/or maintaining the expression of endoderm GRN components by the beginning of gastrulation.

### The AP Wnt signaling network regulates *wnt16* expression and its role in the specification of endoderm GRN components

**Fig. 5.**
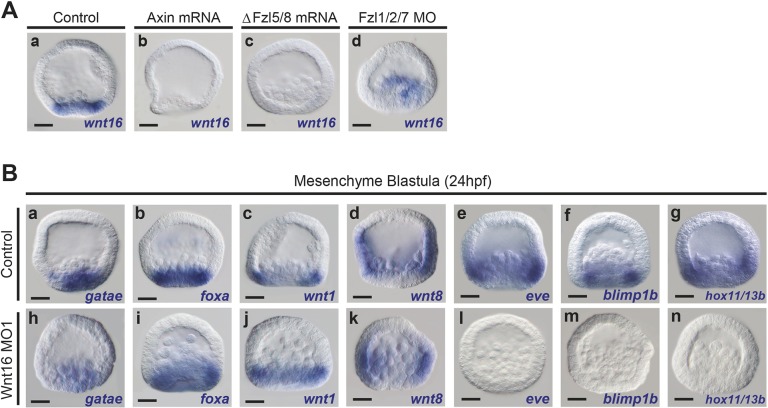
**Regulation of *wnt16* expression by the AP Wnt signaling network and the role of Wnt16 in activating endoderm genes.** (Aa) Control embryo showing *wnt16* expression in the posterior endoderm and mesoderm regions of the embryo at mesenchyme blastula stage (24 hpf). *wnt16* expression was downregulated in Axin mRNA-injected embryos (Ab). *wnt16* expression was downregulated in embryos injected with a dominant-negative form of Fzl5/8 (ΔFzl5/8) (Ac). *wnt16* expression was unperturbed in Fzl1/2/7 morphants (Ad). (B) Wnt16 knockdown embryos at mesenchyme blastula stage showing that Wnt16 was not necessary for the expression of the endoderm genes *gataE*, *foxA*, *wnt1* and *wnt8* (Bh-k compared with Ba-d), but was necessary for the expression of *eve*, *blimp1b* and *hox11/13b* (Bl-n compared with Be-g)*.* MO, morpholino; ΔFzl5/8, dominant negative Fzl5/8. Scale bars: 20 µm.

The zygotic expression profile of Wnt16, with *wnt16* expression in the endoderm and mesoderm regions, suggests that the Wnt signaling network could control its expression. To test this idea, we used morpholino knockdowns or injected mRNA to perturb each of the three Wnt signaling branches. Embryos injected with mRNA encoding Axin, which prevents nuclearization of β-catenin, showed a severe reduction of *wnt16* expression at mesenchyme blastula stage (Fig. 5Ab). Similarly, *wnt16* expression was downregulated in embryos injected ΔFzl5/8 mRNA at the same developmental stage (Fig. 5Ac; Fig. S1C). In contrast, when we perturbed Fzl1/2/7 signaling, *wnt16* expression appeared similar to that in control embryos (compare Fig. 5Aa with Ad). These analyses demonstrate that two of the three signaling branches in the Wnt network are crucial for zygotic *wnt16* expression in the posterior cells that are about to undergo gastrulation at the mesenchyme blastula stage.

Fzl5/8 signaling is necessary for the morphogenetic movements of gastrulation (Croce et al., 2006b) and the activation of Wnt16 by Fzl5/8 in posterior cells correlates with a role in the endodermal GRN governing this process. In addition, a recent study suggested that crucial components of the anterior Veg1 and posterior Veg2 endoderm GRNs (*eve*, *blimp1b* and *hox11/13b*) were slightly downregulated in the absence of Wnt16 (Cui et al., 2014). Therefore, we examined the role of Wnt16 in the activation and/or maintenance of the endoderm GRN at the beginning of gastrulation. Several endoderm GRN components (*gataE*, *foxA*, *wnt1* and *wnt8*) were expressed normally in Wnt16 knockdown embryos (compare Fig. 5Bh-k with Ba-d). However, the expression of *eve*, *blimp1b* and *hox11/13b* was downregulated in Wnt16 knockdown embryos, consistent with the observations in Cui et al. (2014) (Fig. 5Bl-n compared with Be-g). Together, these data suggest that Wnt16 is necessary for the activation and/or maintenance of specific Veg1 and Veg2 endoderm GRN components downstream of Fzl5/8 signaling during gastrulation.

### The function of Wnt16 in the morphogenetic movements during gastrulation and mesoderm morphogenesis

**Fig. 6.**
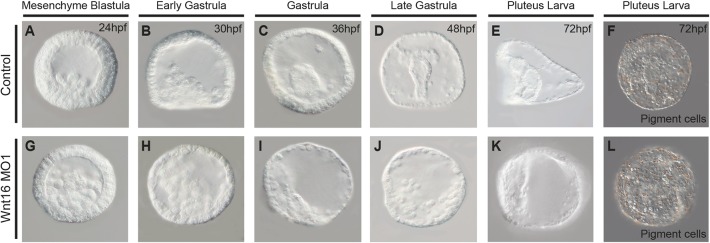
**The role of Wnt16 in morphogenetic movements during gastrulation.** Morphology of control embryos at mesenchyme blastula (24 hpf), early gastrula (30 hpf), gastrula (36 hpf), late gastrula (48 hpf), and pluteus larva (72 hpf) stages (A-E), and embryos injected with morpholino targeting *wnt16* transcripts (G-K). Normal numbers of pigment cells were shown in both control and Wnt16 morphants at pluteus larva stage (F,L), whereas the arrangement of those cells was disrupted in Wnt16 knockdowns. Control=74.5±6.7 (*n*=21); Wnt16 MO1=71±7.7 (*n*=21). Data are mean±s.d. hpf, hours post-fertilization.

The experiments above showing the activation of Wnt16 by Fzl5/8 signaling suggested that Wnt16 might be necessary for the morphogenetic movements involved in gastrulation. Therefore, we observed the morphological phenotypes of embryos injected with Wnt16 morpholino at several stages of development. Cleavage occurred on schedule and embryos developed normally until the mesenchyme blastula stages (Fig. 6G). At this stage, we observed unorganized mesodermal cells in the blastocoel in Wnt16 morphants (compare Fig. 6G with Fig. 6A). During gastrula stages, invagination of the gut was severely disrupted in Wnt16 morphants (Fig. 6H-J versus 6B-D). By pluteus stages, the gut was small and often partially exogastrulated (compare Fig. 6E with 6K). In addition, Wnt16 morphants did not form a skeleton. These phenotypes are similar to those we have observed in Fzl1/2/7 morphants at the pluteus larva stage (Range et al., 2013).

**Fig. 7.**
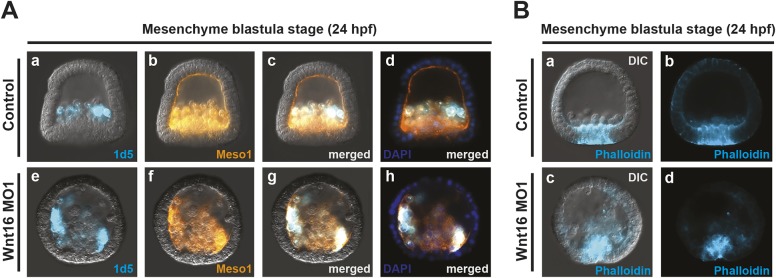
**Wnt16 function in mesoderm morphogenesis and gastrulation.** (A) 1d5 and Meso1 antibody staining at mesenchyme blastula stage (24 hpf). 1d5 (blue) stains skeletogenic mesoderm cells, and Meso1 (orange) is a general mesoderm marker. Neither 1d5 nor Meso1 staining was affected in Wnt16 knockdown embryos (Ae-h) compared with control embryos (Aa-d). (B) F-actin staining as measured by phalloidin binding. F-actin accumulation was present in the invagination of both control and Wnt16 knockdown embryos (Ba-d). MO, morpholino.

Although it appeared that the mesodermal cells in the blastocoel were disorganized, it is possible that there was an increase in the number of either skeletogenic and/or non-skeletogenic (NSM) mesoderm in Wnt16 knockdown embryos. To test this potential increase, we counted the total number of pigment cells, a subset type of NSM cells. Pluteus stage embryos injected with Wnt16 morpholino did not show an increase in the number of NSM pigment cells produced by the embryo (control=74.5±6.7, *n*=21; Wnt16 MO1=71±7.7, *n*=21), but the arrangement of these cells appeared disrupted (Fig. 6F versus 6L). To determine the identity of the disorganized cells at mesenchyme blastula stage, we co-immunostained embryos with the skeletogenic marker 1d5 and the general mesodermal marker Meso1. 1d5 staining and Meso1 staining suggested that the number of skeletogenic and NSM cells were normal in both control and Wnt16 morphants (Fig. 7Aa-h) but that they were randomly distributed throughout the blastocoel. Finally, to develop a better understanding of how Wnt16 affects the morphogenetic movements of gastrulation, we analyzed the patterns of actin accumulation in the sea urchin embryo. F-actin accumulation measured by phalloidin binding was present in the invagination of both phenotypic control and Wnt16 morpholino injected embryos at mesenchyme blastula stage (Fig. 7Ba-d). Together, these data suggest that Wnt16 does not affect the actin accumulation around the presumptive blastopore or skeletogenic and pigment cell numbers during gastrulation, but that it is involved in the proper arrangement of mesodermal cells in the blastocoel.

## DISCUSSION

The data presented here indicate that the secreted Wnt ligand Wnt16 plays a crucial role in Wnt signaling governing early AP axis specification, patterning and gastrulation. A posterior-to-anterior wave of sequential Wnt/β-catenin and Wnt/JNK signaling is essential for the proper positioning of the earliest GRNs along the AP axis: posterior endoderm and mesodermal GRNs, equatorial ectodermal GRN, and the ANE GRN around the anterior pole (Khadka et al., 2018; Range, 2018; Range et al., 2013, 2017). Importantly, Fzl1/2/7 signaling activity appears to interfere with the level of posterior Wnt/β-catenin signaling as well as the timing and/or level of anterior Wnt/JNK signaling, allowing for the proper positioning of these fundamental embryonic territories (Range, 2018; Range et al., 2013). Here, our data from a series of expression studies, functional perturbations and epistasis experiments strongly suggest that an early role of Wnt16 is to activate the Fzl1/2/7 signaling pathway during this fundamental AP patterning process. Subsequently, we show that two of the Wnt signaling network pathways, Wnt/β-catenin and Fzl5/8-JNK, activate zygotic *wnt16* expression in the endoderm and mesoderm during mid-to-late blastula stages. At this developmental stage, Wnt16 activates a distinct mechanism necessary for the regulation of a specific set of endoderm and mesoderm regulatory factors, and the morphogenetic movements of gastrulation.

Increasing the level of Wnt/β-catenin signaling, either through relieving negative or increasing positive inputs, causes an increase in endomesoderm while also eliminating anterior GRNs during the early embryonic AP axis specification in many metazoan embryos (Angerer et al., 2011; Gaspard et al., 2008; Kiecker and Niehrs, 2001; Lekven et al., 2001; Loh et al., 2016; Nordström et al., 2002; Pani et al., 2012; Petersen and Reddien, 2009; Range et al., 2013). Our previous functional data showed that if the broad signaling activity of Fzl1/2/7 signaling is eliminated in sea urchin embryos, then Wnt/β-catenin signaling reporter gene activity increases by ∼2.5 fold at the 60-cell stage (Range et al., 2013). Also shown was that the expression of *wnt8*, which is activated by Wnt/β-catenin signaling (Minokawa et al., 2005; Wikramanayake et al., 2004), is expanded towards the anterior of early blastula stage embryos (Range et al., 2013). We show here that *wnt16*, like *fzl1/2/7*, is broadly expressed during early cleavage stages and that, similar to Fzl1/2/7 knockdowns, there is also only a modest increase in the expansion of endomesoderm GRN component expression activated by Wnt/β-catenin in the absence of Wnt16. In addition, when we overexpressed Wnt16, the expression of these genes was suppressed but not completely downregulated. Together, these data suggest that Wnt16-Fzl1/2/7 signaling provides negative, but not prohibitive, inputs into posterior Wnt/β-catenin signaling, suggesting that they are not the only factors necessary to maintain the correct level of Wnt/β-catenin signaling along the AP axis. Interestingly, we also show that by the 120-cell stage, *wnt8* expression is expanded towards the anterior in the absence of Wnt16 function. During blastula stages in normal embryos, *wnt8* expression is activated in ectoderm cells where the ANE GRN is downregulated, suggesting that it is driving the Fzl5/8-JNK-mediated downregulation in the equatorial ectoderm. We propose that this precocious ectodermal Wnt8 expression in Wnt16 morphants may drive premature downregulation of the ANE GRN by Wnt/JNK signaling in anterior ectodermal blastomeres.

**Fig. 8.**
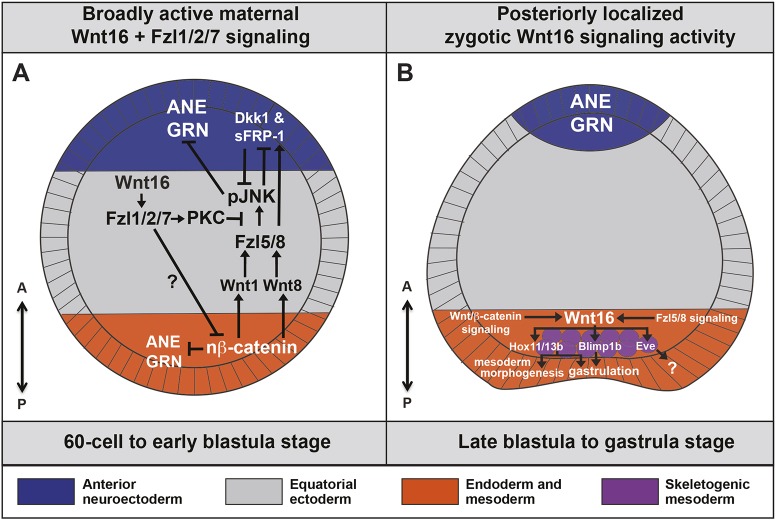
**Model for two phases of Wnt16 activity during early AP axis specification, patterning and morphogenesis of the sea urchin embryo.** (A) Broad maternal non-canonical Wnt16-Fzl1/2/7 signaling antagonizes Wnt/β-catenin and Wnt1/Wnt8-Fzl5/8-JNK signaling during the ANE restriction process. Illustrated is an extended model for early anterior-posterior axis patterning during sea urchin early development (see Introduction and Discussion for details). (B) Posteriorly localized *wnt16* expression in the endoderm and mesoderm territories is activated by Wnt/β-catenin and Fzl5/8 signaling. In addition, the extended model indicates a role for the activation of the key endoderm GRN components, *hox11/13b*, *blimp1* and *eve*. *hox11/13b* appears to be necessary for both gastrulation and mesoderm morphogenesis (Arenas-Mena et al., 2006), and *blimp1b* is necessary for gastrulation (Livi and Davidson, 2006), both similar to the Wnt16 phenotypes described.

As mentioned in the Introduction, an undefined broadly active regulatory mechanism can activate the ANE GRN throughout the sea urchin embryo (Range, 2014, 2018; Range et al., 2013). Wnt/β-catenin signaling in posterior cells and, subsequently, Wnt/JNK in the anterior cells restrict this broad ANE potential to an area around the anterior pole (Range et al., 2013). These pathways are connected by a precisely timed relay mechanism. At the 32-cell stage, Wnt/β-catenin activates Wnt1 and Wnt8 in posterior blastomeres and our functional data suggest that these factors diffuse into anterior cells where they stimulate Fzl5/8-JNK-mediated ANE GRN downregulation as early as the 60-cell stage (Range et al., 2013) (Fig. 8A). In this study, our epistasis experiments strongly suggest that Wnt16 is necessary to activate the Fzl1/2/7 signaling pathway as early as the 32-cell stage to regulate this early phase of ANE restriction, most likely at the intracellular level because knocking down the Fzl1/2/7 receptor produces the same phenotype as the Wnt16 ligand. In a recent study, we also showed that a novel, broadly expressed secreted Frizzled-like protein, sFRP–1, is necessary from the beginning of ANE restriction to antagonize Fzl5/8-JNK signaling (Khadka et al., 2018). Similar to the effect of knocking down Wnt16 or Fzl1/2/7, the ANE GRN is completely downregulated in sFRP-1 morphants during early cleavage stages (Khadka et al., 2018). In addition, overexpression of sFRP–1 prevents the downregulation of the ANE GRN by Fzl5/8-JNK signaling from the anterior half of the embryo (Khadka et al., 2018), mimicking the Wnt16 overexpression phenotype. During early cleavage stages, *wnt16*, *sfrp-1*, *fzl5/8* and *fzl1/2/7* are expressed in the same cells. This overlap suggests that a complex interplay among these factors operates in the extracellular space. Here, it is important to note that our previous functional data indicate that sFRP-1 does not interfere with Wnt16-Fzl1/2/7 signaling. If it did, then embryos injected with sFRP-1 morpholino or mRNA would show opposite phenotypes, i.e. expansion and elimination of the ANE, respectively. Our model proposes that sFRP-1 interferes with Wnt1- and Wnt8-mediated stimulation of Fzl5/8-JNK at the extracellular level (Khadka et al., 2018), but does not interfere with Wnt16-Fzl1/2/7 signaling, which antagonizes the ANE restriction mechanism at the intracellular level. Thus, these two mechanisms work coordinately, but separately, to precisely control the early stages of ANE restriction (Fig. 8A).

As the broadly expressed, maternally supplied *wnt16* mRNA is progressively eliminated from anterior ectodermal cells during early to mid-blastula stages, zygotic *wnt16* expression can be observed around the 120-cell (12 hpf) to mid-blastula stage (15 hpf). Similar to previous studies on zygotic *wnt16* expression that used colorimetric whole-mount *in situ* hybridization in *Strongylocentrotus purpuratus* (Cui et al., 2014) and the Mediterranean sea urchin, *Paracentrotus lividus* (Robert et al., 2014), our analyses showed that posterior endoderm and mesoderm cells express zygotic *wnt16*. Cui et al. (2014) argued that *wnt16* is expressed in the mesodermal territory from 120-cell stage to mid-blastula followed by its expression being cleared from this territory by late blastula stage (18 hpf) when endoderm and mesodermal cells become distinct. *wnt16* is then transiently expressed in a ring of posterior-most endodermal/Veg2 cells where it is downregulated by the beginning of gastrulation (24 hpf) and subsequently upregulated in more anterior Veg1 endodermal cells. They hypothesized that Wnt16 is involved in a posterior-to-anterior inductive cascade from Veg2 mesoderm to Veg2 then Veg1 endoderm that ultimately influences the dynamic expression of *hox11/13b*, *eve*, *blimp1* and *wnt16* (Cui et al., 2014).

Both our colorimetric and more-detailed two-color fluorescence *in situ* hybridization analyses contradict this conclusion. Our results indicate that *wnt16* is broadly expressed throughout the entire endoderm and mesoderm territory at late blastula stage (18 hpf), with the most anterior expression overlapping *eve* expression, which marks the more anterior Veg1 endodermal cells that form the hindgut and midgut. We also observed a downregulation of *wnt16* in the most posterior mesodermal cells by mesenchyme blastula, as did Cui et al., (2014) and Robert et al., (2014). Our results indicate that there is no transient expression and downregulation of *wnt16* from posterior endodermal Veg2 cells to the more anterior Veg1 cells; instead, *wnt16* remains expressed in a concentric ring throughout the Veg1 and Veg2 endodermal cells at mesenchyme blastula stage (24 hpf). Thus, our observations suggest that Wnt16 is not involved in a posterior-to-anterior inductive cascade in the endoderm and mesoderm. Instead, our data indicate that it can act directly on the Veg2 mesodermal GRN in blastulae (12-18 hpf) as well as both Veg1 and Veg2 endodermal cell GRNs from late blastula to mesenchyme blastula stages (18-24 hpf).

To achieve a fuller appreciation of Wnt16 during early development, we also explored the upstream regulation of zygotic *wnt16* expression as well as the downstream GRNs and cellular processes it controls. We show that two of the three AP Wnt signaling network pathways, Wnt/β-catenin and Fzl5/8-JNK, are necessary for the zygotic expression of *wnt16*. Wnt/β-catenin is necessary for the activation of the entire endomesoderm GRN, so it is not surprising that *wnt16* is downregulated. It is interesting that Fzl5/8 signaling is necessary for *wnt16* expression in the endomesoderm. Fzl5/8 signaling has been shown to activate the expression of genes, such as *brachyury*, that are necessary for gastrulation in the sea urchin embryo (Croce et al., 2006b). Similar to embryos in which Fzl5/8 signaling is inhibited, gastrulation does not occur in the absence of Wnt16. These data suggest that Wnt16 may work downstream of Fzl5/8 and may not feedback into this signaling pathway. Instead, it may signal through the three other Frizzled receptors expressed within the same territory by the onset of gastrulation to influence the morphogenetic movements of gastrulation and gut pattern. Consistent with this idea, gastrulation is severely perturbed in Fzl1/2/7 morphants (Range et al., 2013). Given the conservation of many aspects of the early sea urchin endomesoderm GRN among species and the fundamental role of Wnt signaling during early AP specification and patterning, it is important in the future to perform more detailed exploration of the complex interactions among the several Wnt ligands (WntA, Wnt1, Wnt2, Wnt4, Wnt5, Wnt6, Wnt7, Wnt9 and Wnt16, depending on the sea urchin species) and all four Frizzled receptors expressed during the developmental processes leading up to gut patterning and gastrulation.

Downstream of Wnt16, Cui et al., (2014) previously showed that three important endoderm GRN components (*eve*, *hox11/13b* and *blimp1b*) appeared slightly downregulated in the absence of Wnt16. However, embryos showed light expression of these genes in Wnt16 morphants analyzed by whole-mount *in situ* hybridization, suggesting that Wnt16 is only partly necessary for the activity of these genes. Here, we show that all three of these regulatory genes were completely downregulated in our experiments, reinforcing their data. Importantly, both Blimp1b and Hox11/13b knockdowns show severe defects in gastrulation, consistent with a role downstream of Wnt16 (Arenas-Mena et al., 2006; Livi and Davidson, 2006); interestingly, Hox11/13b knockdown embryos show disorganized mesodermal cell populations at mesenchyme blastula stage remarkably similar to those seen in Wnt16 morphants (Arenas-Mena et al., 2006). Again, this is consistent with a role downstream of Wnt16. We analyzed this phenotype in greater detail and our results suggest that these cells are primarily mesodermal and that there does not appear to be an obvious increase in either skeletal or non-skeletogenic mesoderm cells. Interestingly, the larval skeleton does not form in Wnt16 morphants. Wnt16 does not appear to be necessary for the specification of the Veg1 ectoderm GRN (Cui et al., 2014), which is necessary for proper arrangement of the larval skeleton (Duloquin et al., 2007). Thus, we prefer the hypothesis put forth by Arenas-Mena et al. (2006) that cells lacking Hox11/13b could have cell adhesion defects that prevent proper interactions between the mesodermal cells and/or the ectoderm. Alternatively, it could be that these cells have defects in uncharacterized cell-to-cell signaling pathways that are important for correct cellular migration and/or interactions among themselves as well as the ectoderm. Finally, we tested the idea that Wnt16 and/or the GRNs it activates may affect cytoskeletal rearrangements necessary for gastrulation (Beane et al., 2006); however, our data suggest that Wnt16 affects the directed cellular movements of gastrulation by an unknown mechanism. Together, these data give us a better, but still incomplete, picture of the role(s) of Wnt16 during the early stages of gastrulation. In the future, it will be interesting to identify more intermediate and terminal GRN components in endoderm downstream of Wnt16 to determine the cellular and molecular mechanism(s) that are necessary for gastrulation and mesodermal cell behavior.

Relatively few studies have been performed on the role of Wnt16 during embryonic development, and all have been performed in vertebrate species (Fokina and Frolova, 2006; Movérare-Skrtic et al., 2014; Nalesso et al., 2017). As with most Wnt ligands, Wnt16 is involved in multiple developmental processes, and it can stimulate canonical Wnt/β-catenin as well as non-canonical Wnt signaling. For example, Wnt16 is expressed in the ciliary margin zone of the chicken embryo and works to maintain retinal progenitor cells in an undifferentiated state through a Wnt/β-catenin-dependent pathway (Fokina and Frolova, 2006). A recent set of interesting studies in *Xenopus* embryos illustrates the complex nature of Wnt16 and Wnt signaling in general. Movérare-Skrtic et al. (2014) showed that during chondrocyte development, Wnt16 acts as a weak activator of Wnt/β-catenin. Nalesso et al. (2017) then showed that Wnt16 can also act as an antagonist of Wnt/β-catenin if other Wnt proteins are administered, and that this mechanism works through non-canonical signaling. These studies illustrate the multifunctional roles of Wnt ligands, often in the same cells and territories, and also show that Wnt16 can activate a non-canonical pathway that antagonizes Wnt/β-catenin signaling similar to its role in early AP axis specification in the sea urchin (Fokina and Frolova, 2006; Movérare-Skrtic et al., 2014; Nalesso et al., 2017).

In chordates, there are no studies to our knowledge that focus on a role for Wnt16 during early AP axis specification and patterning. However, studies have been performed using qPCR and/or whole-mount *in situ* hybridization in invertebrate deuterostomes to analyze the spatiotemporal expression of Wnt and Frizzled gene expression during this fundamental developmental process. The spatiotemporal *wnt16* expression pattern in these embryos is remarkably similar to that in sea urchin embryos. Although *wnt16* is not maternally expressed in sea star embryos, a low level of *wnt16* expression was observed at the hatched blastula stage similar to sea urchins (McCauley et al., 2013). In addition, *wnt16* expression is upregulated in the endomesoderm during late blastula/gastrula stages (McCauley et al., 2013). Similar to sea stars, *wnt16* is not expressed maternally in hemichordates, but low levels are observed throughout the embryo around blastula stages. At gastrula stages, higher expression levels are localized around the exterior edge of the blastopore lip (Darras et al., 2018). Although Wnt16 is not maternally provided in either sea stars or hemichordates, its early broad expression suggests that it could still play a role in early AP axis specification in these embryos. Importantly, ANE GRN restriction appears to initiate during the blastula stages in each of these species, as opposed to early cleavage stages in sea urchin embryos (Darras et al., 2018; Pani et al., 2012; Range, 2014; Yankura et al., 2013). In addition, *fzl1/2/7* is expressed maternally and broadly expressed throughout the cleavage and early blastula stages in these embryos (McCauley et al., 2013; Range, 2014; Yankura et al., 2013). Based on these studies, it is tempting to speculate that Wnt16-Fzl1/2/7 signaling may play a similar role in AP patterning and/or gastrulation movements in these organisms.

## MATERIALS AND METHODS

### Animals and embryo cultures

Adult *Strongylocentrotus purpuratus* sea urchins were obtained from Monterey Abalone Company (Monterey, CA, USA), Marinus Scientific (Longbeach, CA, USA) and the California Institute of Technology (Pasadena, CA, USA). The gametes were collected by injecting 0.5 M KCl into the body cavity of adult sea urchins. Fertilized embryos were cultured at 15°C in artificial seawater (ASW).

### RNA extraction and cDNA clone preparation

RNA from embryos at different times of development was extracted and purified using RNeasy Plus Mini kit (Qiagen). Purified RNA was reverse transcribed to cDNA using SuperScript IV First-Strand Synthesis System (Invitrogen) and random primers for RT-PCR. Total RNA samples for qPCR were treated with DNase I from the DNA-free kit (Invitrogen) to remove any possible residual genomic DNA. cDNA from 24 hpf mesenchyme blastula stage embryos was used to obtain full-length clones for *wnt16*. The following primers, based on the sea urchin genome sequence, were used to insert *wnt16* into pGEMT-easy and pCS2+ for *in situ* probe and mRNA synthesis, respectively: *in situ* forward, 5′-ATATCATGGACTGCGGACTA; *in situ* reverse, 3′-GTCCATGGTTTAAGCAGACC; pCS2+ vector forward, 5′-CGCGGATCCACCATGGAGTGTAGCAAT; pCS2+ vector reverse 3′-CCGCTCGAGTCATTTACAAGTGTAGAT.

### mRNA overexpression and morpholino injections

For overexpression studies, the Wnt16-pCS2+, ΔFzl5/8-pCS2+ and Axin-pCS2+ vectors were linearized with NotI restriction enzyme. mRNA was synthesized using the SP6 mMessage Machine kit (Ambion) according to the manufacturer's protocol, further purified by LiCl precipitation and injected into the embryos at the following concentrations: ΔFzl5/8 mRNA, 1.0 μg/μl; *wnt1* mRNA, 0.05 μg/μl; *wnt8* mRNA, 0.65 μg/μl; *wnt16* mRNA, 0.5 μg/μl; *axin* mRNA 1.5-2.0 μg/μl.

*S. purpuratus* EST, genomic and cloned *wnt16* sequence was used to generate translation-blocking morpholino-substituted oligonucleotides 2 (MO2) (Gene-Tools). The sequences and injection concentrations of all the morpholino oligomers were as follows: Fzl1/2/7 MO, 5′-CATCTTCTAACCGTATATCTTCTGC-3′ (1.3 mM) (Range et al., 2013); Wnt16 MO1, 5′-TCTCAACAAACTCGATAGTTCAACC-3′ (0.8 mM) (Cui et al., 2014); Wnt16 MO2, 5′-CAAAACATCGGTAGCTTAAATCCAT-3′ (0.35 mM); and Wnt7 splice MO, 5′-TCCTCGTCGTATATCCTTACCAGCA-3′ (1.5 mM).

As a control for morphological and developmental defects related to injections, we used a standard control morpholino: 5′-CCTCTTACCTCAGTTACAATTTATA-3′ from Gene-Tools. Standard control and experimental morpholinos were injected at the same concentrations. For mRNA and morpholino microinjections, eggs were de-jellied by passing them through 74 μm mesh Nitex, plated in rows on a culture dish coated with 25% protamine sulfate and fertilized with diluted sperm. After fertilization, embryos were immediately injected with 15% FITC (2.5 μg/ml), 20% glycerol and mRNA and/or morpholino oligonucleotides, and cultured at 15°C until the desired developmental time. For each mRNA or morpholino injection experiment, 50-200 embryos from at least three batches of different mating pairs were used. Only experiments with changes in phenotype or marker expression in at least 85-90% of the injected embryos were considered conclusive.

### Quantitative polymerase chain reaction

Quantitative polymerase chain reaction (qPCR) assays were performed as described previously (Wei et al., 2009). Each qPCR experiment was repeated with embryos from at least three different mating pairs and each PCR reaction was carried out in triplicate for each developmental stage. The qPCR primer set information for ANE GRN is from Range et al. (2013). The endomesodermal and Wnt16 primers are included in Table S1. To calculate the developmental expression levels of *wnt16*, the number of transcripts per embryo was estimated based on the ΔCt value of the z12 transcript (Range et al., 2013; Wang et al., 1995). To compare differential expression between control and perturbed embryos, mitochondrial 12 s RNA ΔCt values were used to normalize the relative target gene expression levels. In differential gene expression, a twofold or higher change in expression level was considered to be significant.

### Whole-mount *in situ* hybridization

Antisense RNA probes, complementary to the target mRNA, for each gene analyzed were synthesized from linearized pGEM-T Easy or pCS2+ plasmids using T7 or SP6 polymerase enzyme. Alkaline phosphatase reporter and two-color fluorescent *in situ* hybridization were carried out as previously described (Wei et al., 2009; Sethi et al., 2014). For the two-color *in situ* hybridization (Fig. 4), *wnt16* was labeled with DIG and detected with fluorescein-TSA and *eve*, *foxA* and *gcm* were labeled with fluorescein and detected with Cy3-TSA.

### Immunohistochemistry

Embryos were fixed in 2% paraformaldehyde in artificial seawater for 20 min at room temperature. For 1d5 and Meso1 staining, embryos were washed five times in phosphate-buffered saline containing 0.1% Tween 20 (PBST). Embryos were incubated at 4°C overnight with primary antibodies against 1d5 (1:25) and Meso1 (1:50) in PBST and 4% normal goat serum. Primary antibodies were detected by incubating embryos for 1 h at room temperature with 1:1000 Alexa Fluor-coupled 488 goat anti-mouse IgM and 1:1000 Alexa Fluor-coupled 555 goat anti-mouse IgG secondary antibodies (Thermo Fisher Scientific). For actin/phalloidin staining, embryos were washed three times through phosphate-buffered saline containing 0.1% Triton X-100 and kept overnight at 4°C. Embryos were incubated with 1:2000 Alexa Fluor 488 Phalloidin (Thermo Fisher Scientific) in 3% BSA in PBST 0.1% Triton overnight at 4°C. Nuclei were stained with DAPI (1:3000).

## Supplementary Material

Supplementary information
