# Supplementary material for: A biphasic role of non-canonical Wnt16 signaling during early anterior-posterior patterning and morphogenesis of the sea urchin embryo
Source: Development. 2019 Dec 16;146(24):dev168799. doi: 10.1242/dev.168799 (PMC6955209; doi:10.1242/dev.168799)
Supplement: Supplementary information [file develop-146-168799-s1.pdf]

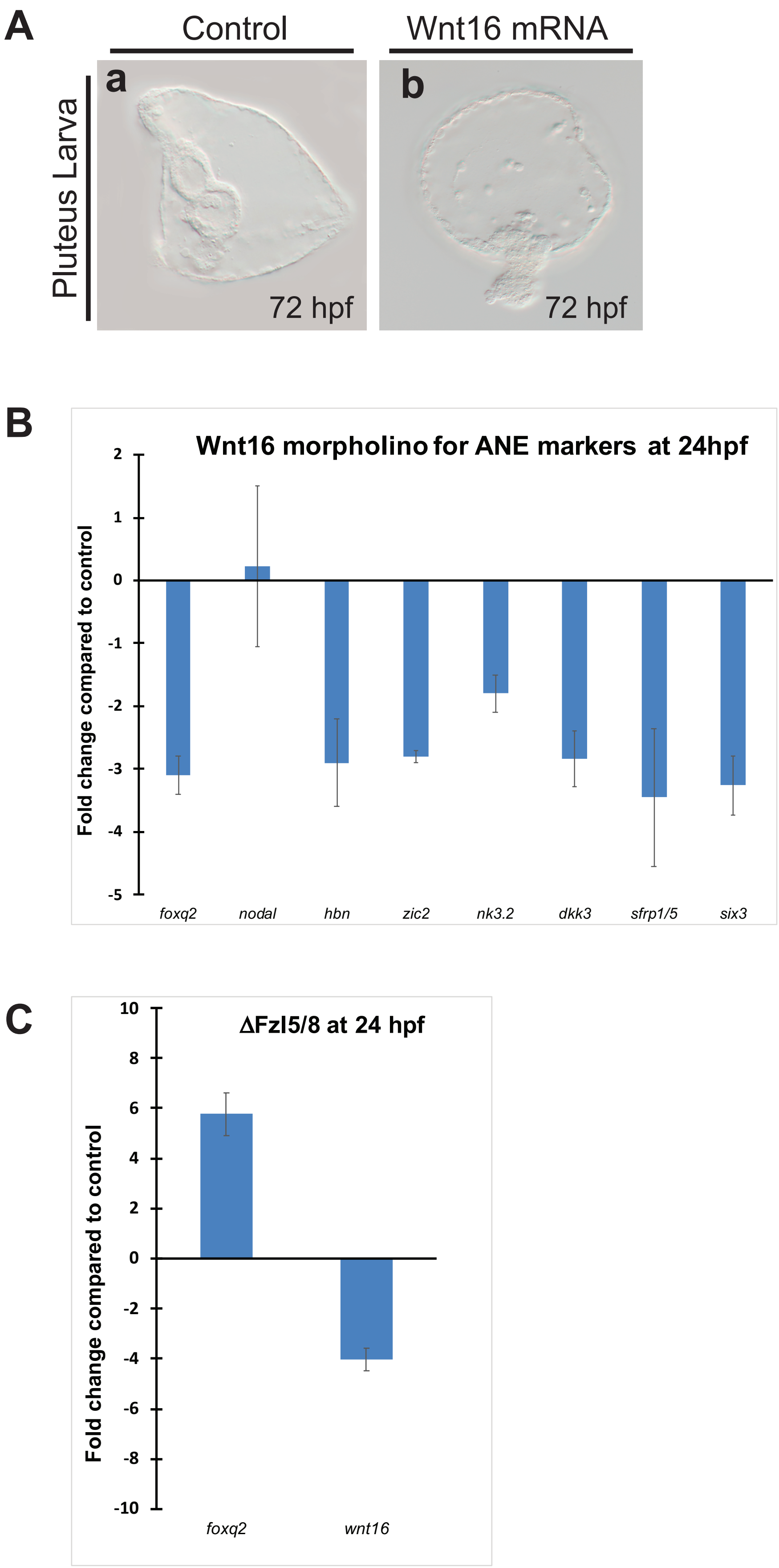
A
Control
Wnt16 mRNA
Pluteus Larva
a
b
72 hpf
72 hpf
B
Wnt16 morpholino for ANE markers at 24hpf
Fold change compared to control
2
1
0
-1
-2
-3
-4
-5
foxq2
nodal
hbn
zic2
nk3.2
dkk3
sfrp1/5
six3
C
ΔFzl5/8 at 24 hpf
Fold change compared to control
10
8
6
4
2
0
-2
-4
-6
-8
-10
foxq2
wnt16

Figure S1. Wnt16 overexpression phenotype and qPCR analysis for ANE GRN members and *wnt16* expression. (A) Phenotypes for control and Wnt16 overexpression at late gastrula stage. (B) qPCR analysis from three different batches of embryos showing the downregulation of ANE regulatory factors at mesenchyme blastula stage (24 hpf) in Wnt16 knockdowns. (C) qPCR data showing that *wnt16* expression was downregulated in embryos injected with a dominant negative form of Fzl5/8 (ΔFzl5/8) at mesenchyme blastula stage (24 hpf). The *y*-axis shows the fold change in gene expression level in Wnt16 knockdowns and *ΔFzl5/8* mRNA injected embryos compared to control embryos at mesenchyme blastula stage (24 hpf). MO, morpholino; Scale bars: 20 µm.

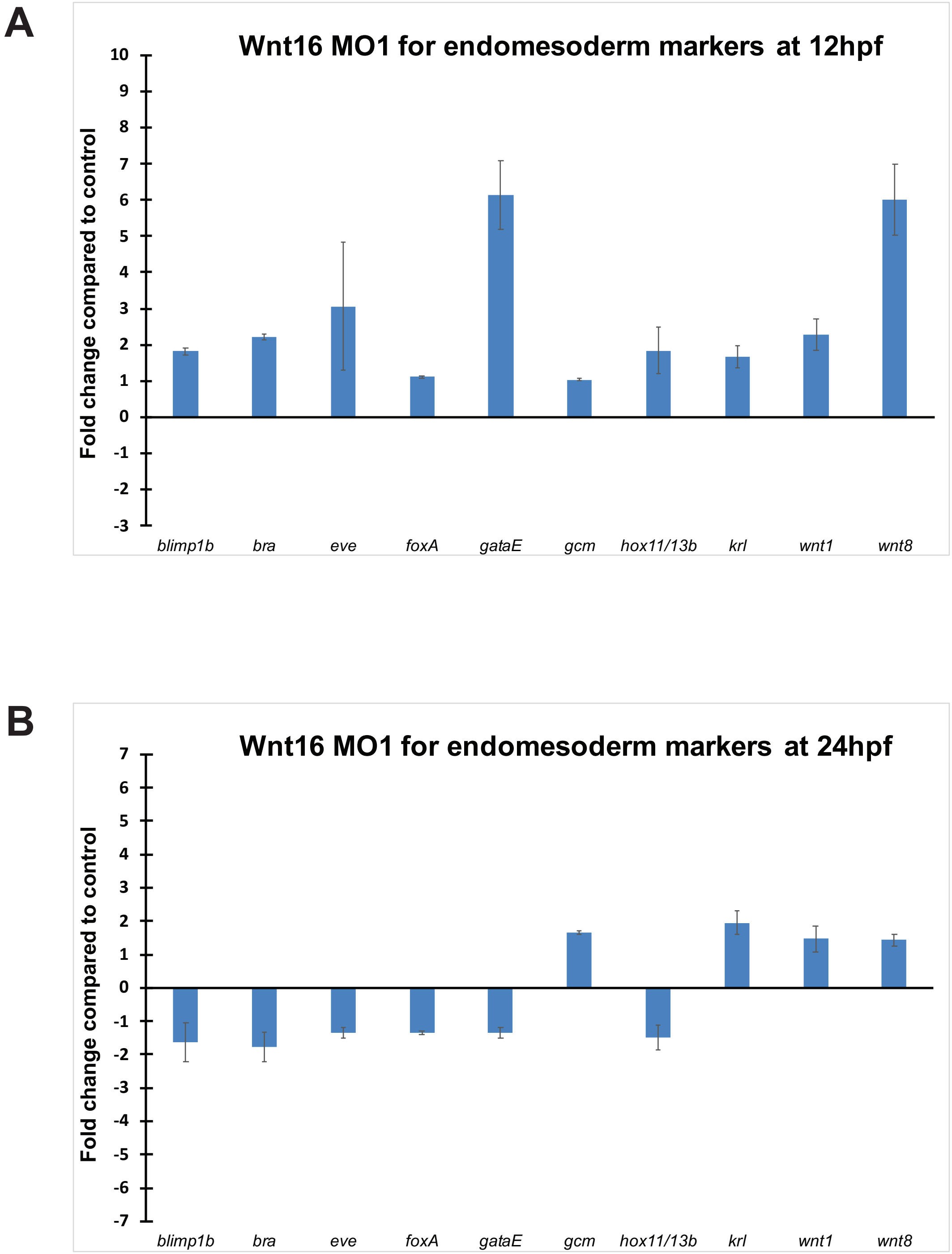
A
Wnt16 MO1 for endomesoderm markers at 12hpf
Fold change compared to control
10
9
8
7
6
5
4
3
2
1
0
-1
-2
-3
blimp1b
bra
eve
foxA
gataE
gcm
hox11/13b
krl
wnt1
wnt8
B
Wnt16 MO1 for endomesoderm markers at 24hpf
Fold change compared to control
7
6
5
4
3
2
1
0
-1
-2
-3
-4
-5
-6
-7
blimp1b
bra
eve
foxA
gataE
gcm
hox11/13b
krl
wnt1
wnt8

Figure S2. qPCR analysis for endomesoderm GRN factors.

(A) qPCR measurements from three different batches of embryos showing the upregulation of several endomesoderm factors at 120-cell stage (12 hpf) in Wnt16 knockdowns. (B) qPCR measurements from three different batches of embryos showing that the expression of endomesoderm makers was not affected in Wnt16 morphants at mesenchyme blastula stage (24 hpf). The *y*-axis shows the fold change in gene expression level in Wnt16 knockdowns compared to control embryos at mesenchyme blastula (24 hpf) and 120-cell (12 hpf) stages. MO, morpholino; Scale bars: 20 μm.

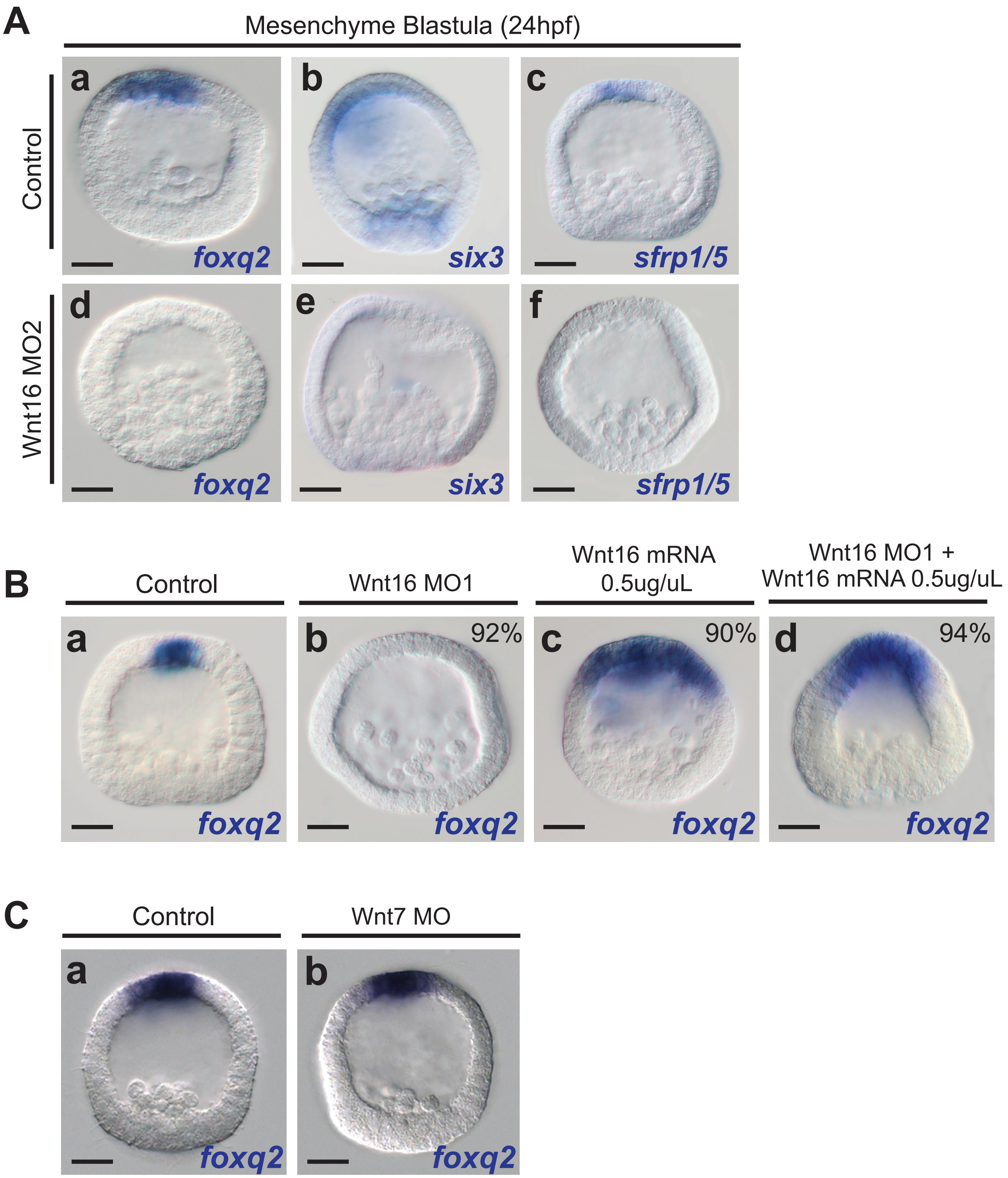
A
Mesenchyme Blastula (24hpf)
Control
Wnt16 MO2
a
b
c
d
e
f
foxq2
six3
sfrp1/5
foxq2
six3
sfrp1/5
B
Control
Wnt16 MO1
Wnt16 mRNA 0.5ug/uL
Wnt16 MO1 + Wnt16 mRNA 0.5ug/uL
a
b
92%
c
90%
d
94%
foxq2
foxq2
foxq2
foxq2
C
Control
Wnt7 MO
a
b
foxq2
foxq2

Figure S3. Additional morpholino phenotypes and controls. (A) The ANE makers are downregulated in embryos injected with Wnt16 MO2 (Ad-f) compared with control embryos (Aa-c). (B) Expression of the ANE marker *foxq2* in control (Ba), Wnt16 MO1-injected (92%; *n*=68/74; Bb), and *wnt16* mRNA-injected embryos (90%; *n*=54/60; Bc) at mesenchyme blastula stage. Wnt16 overexpression rescues *foxq2* expression in embryos injected with Wnt16 MO1 (94%; *n*=64/68; Bd), which cannot bind *wnt16* mRNA. (C) *foxq2* expression was unperturbed in embryos injected with Wnt7 morpholino. MO, morpholino; Scale bars: 20 μm.

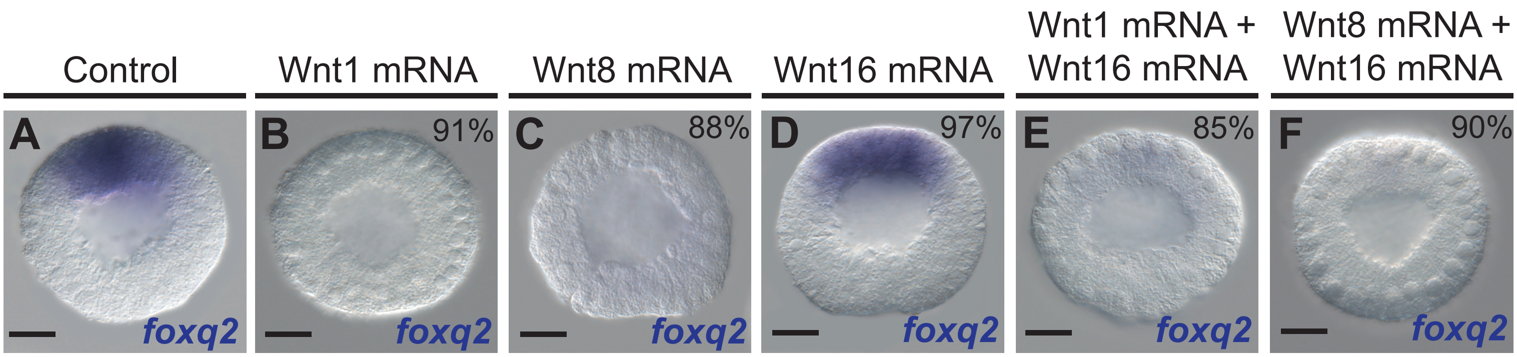

Figure S4. Expression of the ANE marker *foxq2* in embryos co-injected with *wnt16* mRNA and *wnt1* or *wnt8* mRNA at 120-cell stage. The expression of the ANE marker *foxq2* was downregulated in embryos overexpressing *wnt1* (91%) and *wnt8* mRNA (88%) at 120-cell stage (B and C). *foxq2* was expressed in embryos injected with *wnt16* mRNA (97%) (D). In addition, double injected embryos with *wnt16* mRNA and either *wnt1* (85%) (E) or *wnt8* mRNA (90%) (F) downregulated the expression of *foxq2*. Scale bars: 20 μm.

Table S1. qPCR primers pairs used for expression analysis.

Sp-mitochondrial 12s rRNA	Forward 5’-ACTCTCTCCTCGGAGCTATA-3’<br>Reverse 5’-GTATAATTTTTGCGTATTCGGC-3’
Sp-zic2	Forward 5’-GAGGGATGTGATCGTCGTTT-3’<br>Reverse 5’-ACTGCTGTCGTTGGCTTCTT-3’
Sp- blimp1b	Forward 5’-TCGCTATGCGGGATCTCTAC-3’<br>Reverse 5’-GGGGTCCTTGACCTCGTAA-3’
Sp-bra	Forward 5’-ACACATCGACCCATCATCAA-3’<br>Reverse 5’-CATGGTGTCGTATCTTGGAAAG-3’
Sp-eve	Forward 5’-CACAGACCCTGGACTTTCGT-3’<br>Reverse 5’-GACAAACGGTCATCCCACTT-3’
Sp-foxA	Forward 5’-CCAACCGACTCCGTATCATC-3’<br>Reverse 5’-CGTAGCTGCTCATGCTGTGT-3’
Sp-gataE	Forward 5’-CTGGCTCAAGACGAGAAGGA-3’<br>Reverse 5’-CCTCTTCCGAGTCTGAATGC-3’
Sp-gcm	Forward 5’-CGACTGATAACCACGCTCAA-3’<br>Reverse 5’-TTAACGACGTCGGTCGATTC-3’
Sp-hox11/13b	Forward 5’-CACAGGCTCTCGACCTAACC-3’<br>Reverse 5’-GGTGGATGAGGTGGTAGATGA-3’
Sp-krl	Forward 5’-CACGAACTCTTCGCAATCAA-3’<br>Reverse 5’-CCAAGGGACAGGAGTGAAGA-3’
Sp-wnt1	Forward 5’-ACCTTCAGAACGGTGGGTGA-3’<br>Reverse 5’-AGCTTGATTCGGTTTCGCG-3’
Sp-wnt8	Forward 5’-TGTCGTTCATTCAAGCCATC-3’<br>Reverse 5’-TATCACTCGCCATTCGTTCA-3’
Sp-wnt16	Forward 5’-CTTACCGAATGTGGATGTGC-3’<br>Reverse 5’-TATCGCCAGAGTCGACAAAC-3’

Development • Supplementary information
